# Relationship between green entrepreneurship orientation, integration of opportunity and resource capacities and sustainable competitive advantage

**DOI:** 10.3389/fpsyg.2022.1068734

**Published:** 2022-12-19

**Authors:** Wenjin Li, Yu Sun, Yang Gao

**Affiliations:** ^1^School of Applied Mathematics, Jilin University of Finance and Economics, Changchun, China; ^2^School of Economics and Management, Dalian University of Technology, Dalian, China; ^3^Management School, Hainan University, Haikou, China

**Keywords:** green entrepreneurship, opportunity, resource, capabilities, sustainable competitive advantage

## Abstract

Green entrepreneurial orientation (GEO) and sustainability have been the hot topics in green entrepreneurship research. The GEO integrates market orientation (MO) and environmental orientation (EO), and enterprises need to increase resource investment. However, it is not clear whether this strategic orientation can help new ventures achieve sustainable competitive advantages (SCA). In addition, the integrated role of opportunities and resources in the process of green entrepreneurship cannot be ignored. To fill the gap, we analyzed survey data from 274 emerging green enterprises in China, and we examined the effect of GEO on SCA. Additionally, we proposed the concept of integration of opportunity and resources capabilities (IORC) by integrating the core factors of green entrepreneurship. It can be divided into internal integration (ITI) and external integration (ETI), and the moderating effect of IORC on this relationship was also inspected. The results indicated that GEO significantly affects SCA, and IORC positively moderates the relationships between GEO and SCA. Indeed, compared with the enterprises that adopt a single strategic orientation, enterprises that adopt GEO can obtain greater SCA. In addition, focusing on the internal and external integration of IORC can further enhance the acquisition of SCA. This study not only integrates the theories of entrepreneurship and sustainable development but also compensates for the lack of green entrepreneurship theory while providing practical guidance for new enterprises seeking to engage in green entrepreneurship.

## Introduction

The global economy develops rapidly, but the environment has become increasingly degraded. The contradiction between environment and economy development has become a difficult problem in theory and practice. Green entrepreneurship has gradually attracted the attention of scholars concerned about increasingly degraded natural environment (Garcés-Ayerbe et al., 2022; Sun et al., 2022). More and more countries and governments regard green entrepreneurship as an important means to address environmental problems while achieving sustainable economic development (Zhao et al., 2021; Suki et al., 2022). Green entrepreneurship has also become a new value creation process that enables enterprises to respond to social and ecological exigencies while developing the economy.

Green entrepreneurship not only integrates two important theories of business entrepreneurship and sustainable development but also plays a vital role in environmental, economic, and social sustainability (Kraus et al., 2018; Le Loarne-Lemaire et al., 2022). However, since green entrepreneurship involves the compound behavior of two fields, it requires different strategic attributes and a different strategic posture. That is, enterprises engaged in green entrepreneurship must possess the unique characteristics of a startup enterprise as well as the ability to pursue environmental and sustainable development (Ramadani et al., 2022; Theodoraki et al., 2022). Thus, the premise of the effective development of green entrepreneurial activities based on “the economy” and “the environment” is that enterprises have a dual composite orientation, that is, a green entrepreneurship orientation (GEO). However, the existing studies focus more on the impact of a single strategic orientation or corporate competitive advantage, while research focused on a composite orientation and sustainable competitive advantage (SCA) in different fields remains rare. Particularly in the area of green entrepreneurship, the research on GEO and corporate sustainable competitive advantage has a certain value.

The nature of green start-ups remains entrepreneurship. The essence of entrepreneurship is to realize the maximum value of opportunity under resource constraints (Jiang et al., 2018). Therefore, start-ups must follow the core rules of entrepreneurship and attach importance to matching the two core elements of opportunity and resources (Li et al., 2021). There have been many studies on how to match these elements from a single perspective of either opportunity or resources. However, few of these studies adopt a systematic perspective. That is, entrepreneurial opportunity and resources are linked and integrated, and the interaction between them constitutes a complete and independent entity. From a systematic perspective, it is important to integrate opportunities and resources to reveal the essential nature of entrepreneurship.

This study is guided by the following research question: whether the new enterprises with GEO as a strategic orientation can gain SCA and whether the relationship between the two is associated with IORC. In answering this question, we explored the relationship between GEO and SCA using survey data from 274 emerging green enterprises in China. Then, we innovatively proposed the concept of integration of opportunity and resources capabilities (IORC), which not only systematically integrates the two core elements of entrepreneurship but also reveals the essence of entrepreneurship. Finally, we examined the role of IORC in GEO and SCA.

This paper contributes to the theory and practice. First, we integrate the theory of entrepreneurship and the sustainable development theory and reveal the significance and essence of GEO and its relationship with SCA. In this, we compensate for the inadequacy of entrepreneurship theory, which is prone to focus on economic benefit, while promoting the development of GEO theory. Second, the new concept of IORC proposed in this study is of substantial value in revealing the essence of entrepreneurship and improving its success rate. This contribution should be of interest to prospective entrepreneurs. Finally, the paper establishes a complete theoretical model for use in a comprehensive empirical test and systematically investigates issues that green start-ups must pay attention to in the process of pursuing SCA, including adhering to GEO and cultivating IORC.

## Theoretical background and hypotheses

### Green entrepreneurship

Traditionally, in developing countries, when enterprises transform products and services into profits, managers rarely care about the negative impact of their decisions and behaviors on the environment (Shahzad et al., 2021). With the emergence of a large number of ecological and environmental problems, managers were increasingly forced to consider the impact of their organizations on the natural environment (Garcés-Ayerbe et al., 2022). As a result, green management appeared. The emergence of the green economy also represents part of a new social structure (Allen and Malin, 2008). Based on the positive impact of SMEs on the sustainable development of society (Levinsohn, 2013), scholars proposed that greening could also create sustainable opportunities for new enterprises. However, most of the studies on this topic focused on green management institutions (Babiak and Trendafilova, 2011). Relatively few addressed green entrepreneurship. By the 1990s, as ecological values gradually formed, the green market emerged, and economic profit created by the emerging green market linked the interests of entrepreneurship and the social environment, scholars found that being green did not place a burden on the enterprise. It was more likely to enable enterprises to have the opportunity to develop their advantageous resources and positive performance impact (Garcés-Ayerbe et al., 2022). The view that entrepreneurs are not environmentally conscious or not concerned regarding the environment became outdated. Green entrepreneurship has been addressed in terms of the dual role of market orientation and GEO (Makhloufi et al., 2021; Zhou et al., 2021). As a new type of entrepreneurship, green entrepreneurship differs from traditional approaches, and green enterprises differ from traditional ones. They are not only concerned with short-term profit. Green entrepreneurs hope to simultaneously value the ecological environment, the economy, and society (Silajdžić et al., 2015). However, the value placed on the ecological environment is the key to competition. More specifically, the first step is ecological participation. Although the degree of green is not the same, all green enterprises display an essential “tendency to innovate and create green organizations” (Suki et al., 2022). Second, to survive and develop by using green advantages and relying on green markets and green consumers, the most obvious tactic of green entrepreneurship is to exploit the green market as the breakthrough point for entrepreneurship in terms of strategic choice. In terms of product market positioning, the green market is the target market for green entrepreneurship. Without a green market of an initial scale, green entrepreneurship has no basis for survival, and there is no room for development. In addition, green enterprises must make consumers aware of the unsustainability of existing technologies and products and encourage their demand for green products. Finally, long-term periodicity and policy dependence are important (Zhao et al., 2021). Since the return period of green entrepreneurship is long and involves certain social responsibilities, green entrepreneurship is typically generated against the background of policy encouragement or support and often plays the dual role of ecological construction and entrepreneurship. Its core lies in discovering future market opportunities, developing the green market with innovative products, and accepting the ecological responsibility of enterprises.

In the research on green entrepreneurship, scholars have reached consensus on the following three points. First, defined according to its purpose, green entrepreneurship should seek to actively achieve ecological environmental goals by means of entrepreneurship, whereby a green participation tendency is emphasized. Secondly, from the perspective of the development opportunities, green entrepreneurship emphasizes the use of green market opportunities gradually popularized and expanded by green values to open up new markets and create new profit growth points. Finally, from the perspective of the main business of green entrepreneurship, green entrepreneurs primarily open the market and improve the competitiveness of their enterprises by a forward-looking understanding of the future market and developing green products and services that will meet future demand. It can be noted that green entrepreneurship is an entrepreneurial behavior that involves the dual roles of ecological environment orientation and market orientation. From the perspective of society, green entrepreneurship, as a new way of entrepreneurship, can also be understood as a green ecological revolution carried out by an entire society to reform the original business model and improve the economic structure.

### Green entrepreneurial orientation and sustainable competitive advantage

“Orientation” refers to an enterprise’s strategic attitude. Regarding orientation, entrepreneurship scholars have proposed the mature concept of entrepreneurship orientation (Wiklund and Shepherd, 2003). Entrepreneurial orientation (EO) first originated in the categorization of enterprise types but was not known as such. EO refers to the characteristics of entrepreneurial enterprises, mainly including product or technology innovation, the risks enterprises must take, and to a certain extent three types of advanced action. These characteristics are used to distinguish startups and conventional enterprises. Later, EO was defined as “entrepreneurial strategic attitude,” which refers to an overall market competition orientation and organizational behavior tendency of start-ups, including how to adopt competitive and innovative strategies to lead market competitors (Covin and Slevin, 1989). Finally, the concept of EO was proposed and defined as the process and practice of strategic decision-making activities that could trigger new enterprise creation and new entrepreneurial behaviors (Lumpkin and Dess, 2001).

Public awareness of environmental protection, government policies, global standards, and other factors urge enterprises to pay attention to the green strategy (Verma and Kumar, 2021). Where GEO differs from entrepreneurial orientation is that GEO is a strategic posture that combines the dual activities of pursuing a green ecology and market competition (Makhloufi et al., 2021; Zhou et al., 2021). It is a compound orientation that involves interaction between greenness and entrepreneurship, which includes entrepreneurship in the field of environmentalism. The strategic posture also includes ecological and environmental strategic characteristics, that is, acting in a sustainable and green way. It is a composite orientation that integrates entrepreneurship and ecology. GEO implies that venture enterprises not only use market opportunities as the source of entrepreneurship but also intend to “ecological” entrepreneurship. It can be noted that GEO emphasizes both market competition and the ecological environment tendency. MO and EO coexist and interact. Therefore, the understanding of GEO in this paper primarily refers to the interaction between market orientation and environment orientation, which is a composite strategic orientation. Both EO and MO are not completely fragmented in most cases, and most enterprises and entrepreneurs will adopt both. Thus, it is worthwhile to study whether the composite orientation can help the enterprise obtain a larger SCA than a single orientation. In addition, whether there is a mutual promotion between the two orientations is worthy of further study.

Based on the competitive advantage theory, SCA refers to the enterprise can always have an advantage over its competitors. SCA enables enterprises to obtain long-term benefits, which implies creating products or services that cannot be duplicated or imitated by competitors. Some scholars find that resources and capabilities are the keys for enterprises to obtain SCA from the perspective of strategic management (Ge et al., 2018; Makhloufi et al., 2021). In particular, enterprises with dynamic capabilities can act quickly to adapt to environmental changes (Jiang et al., 2018). Moreover, from a sociological perspective, scholars have noted that social responsibility can enable enterprises to obtain SCA (McWilliams and Siegel, 2010), including green management, energy conservation, green product design and production, business ethics and other fields. In this paper, SCA is measured by financial indicators (e.g., the sales growth rate and profit growth rate) and non-financial indicators (e.g., customer satisfaction and loyalty). Before conducting systematic studies, we need to clarify the effects of MO and EO on SCA from a single perspective. MO is defined as the process by which an enterprise realizes its competitive advantage through competition and other means in the target market, reflecting the positioning of the enterprise with respect to customers, competitors and other external factors (Slater et al., 2010). MO enterprises can be sensitive to and aware of the occurrence of events and trends in advance and thus predict events more accurately, retain as well as attract customers, improve channel relations or hinder the actions of competitors. In addition, MO can help technology-based entrepreneurship achieve better performance. Therefore, a high-market-oriented enterprise can quickly explore market opportunities and respond to market demands. Compared with its competitors, such an enterprise does not find it easy to deviate from market rules in the process of starting a business, and it is not easy to separate innovative products from market demands, so it is easier to gain competitive advantages (Kumar et al., 2011). Particularly for developing countries, the high certainty and adaptability of market orientation can compensate for the high risk caused by the current environmental uncertainty and enable enterprises to continuously gain competitive advantages.

EO emphasizes that the enterprise should consider the influence of environment in the process of strategy formulation. It is a strategic attitude at the corporate level. This strategic orientation promotes the sustainable development of the ecological environment by means of, e.g., green product development and technological ecological process reengineering (Dangelico and Pujari, 2010). EO can be divided into endogenous environmental orientation and exogenous environmental orientation. The first refers to the commitment of enterprises to environmental protection based on their own values and ethical standards, which is thus a more endogenous form of self-restraint. In contrast, an exogenous environmental orientation implies that an enterprise’s attitude toward the environment is influenced by the regulatory environment. The focus of this paper is the endogenous environmental orientation. In addition, there is consensus in the academic community regarding whether GEO can enable enterprises to obtain SCA (Chang, 2011). Although in early research, certain scholars believed that economic performance and environmental performance were not equal within enterprises and that paying attention to ecological environment construction was bound to increase enterprise costs and sacrifice economic benefits (Schaltegger and Synnestvedt, 2002). However, with the development of the research and the maturity of the market environment and structure, the persuasiveness of such views had been undermined. Scholars have increasingly come to believe that an environment-oriented strategy that takes into account the ecological environment can increase enterprise competitiveness (Zhou et al., 2021). In particular, mature green products and green production technology systems can enable enterprises to surpass their competitors more readily and make it more difficult for competitors to imitate them. Therefore, enterprises that adopt environmental orientation can gain sustainable competitive advantage.

Based on this reasoning, this paper proposes that GEO is a composite orientation of market-oriented and environment-oriented interaction and helps enterprises obtain higher SCA. We propose the following three hypotheses.

*H1*: Enterprises with higher levels of MO has a positive effect on SCA.

*H2*: Enterprises with higher levels of EO has a positive effect on SCA.

*H3*: Enterprises with higher levels of GEO has a positive effect on SCA.

### Integration of opportunity and resource capabilities

**Figure 1:**
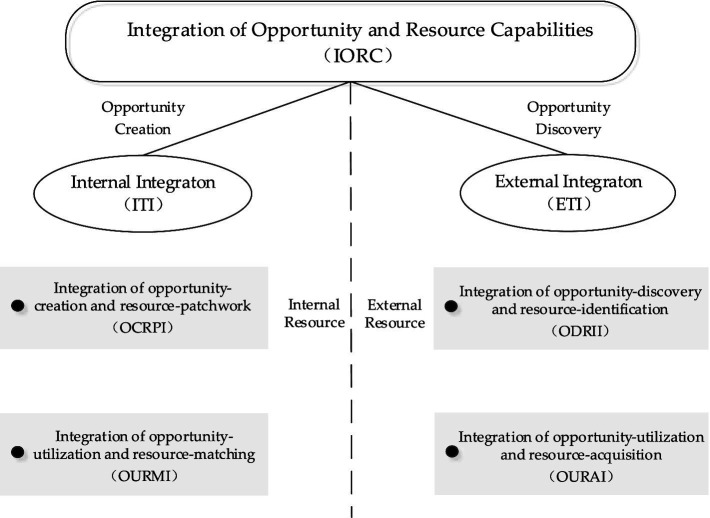
The model of the IORC.

The relationship between opportunities and resources is complex, but the research on them is fragmented. The introduction of the IORC helps to clarify the connection between opportunities and resources, and makes them form an “integration effect” in the process of entrepreneurship (Li et al., 2021). Reviewing the existing research, Shane and Venkataraman (2000) argued that the essence of entrepreneurial behavior is opportunity identification and opportunity utilization Brown et al. (2001) and Baker and Nelson (2005), pointed out that resource development can solve the resource constraints in the early stage of entrepreneurship, and Ge et al. (2016) outlined the connotation of “the opportunity in resource” and “the resource in opportunity” from a systematic perspective. Based on this, this paper takes internal and external resources as the dividing line and divides the interaction between resources and opportunities into two parts (Li et al., 2021): internal integration (ITI) and external integration (ETI), as shown in Figure 1.

First, ITI and ETI are indispensable. This paper refers to the *Yin-Yang balance* theory as the classification principle of ITI and ETI. This theory comes from the dialectical materialism in traditional Chinese culture. It argues that the existence and development of things include two sides: “Yin” and “Yang.” The relationship between the two is opposite and mutual. The opposite relationship is not static, but relative and dynamic. The balance between the two needs to be maintained (Li, 2012). On this basis, this paper introduces the theory of *Yin-Yang balance* to explain the process of entrepreneurship. ITI and ETI are distinguished based on the internal and external properties of the resource. ITI focuses on internal resource and opportunity creation, which means that enterprises create opportunities through resource patchwork and seize opportunities through resource integration (Li et al., 2021). ETI, on the other hand, emphasizes the role of external resource acquisition and opportunity discovery. ITI and ETI are two key elements of entrepreneurship, one is “Yin” and the other is “Yang.” Enterprises need to make efforts in two aspects to balance the entrepreneurial system (Wiklund and Shepherd, 2003).

Second, ITI and ETI are effective. ITI is divided into two sub-dimensions. One is the integration of opportunity creation and resource patchwork, which means creating opportunities by piecing together the resources at hand. The other is the integration of opportunity utilization and resource-matching, which means developing opportunities by integrating resources. In the early stage of entrepreneurship, faced with the constraint of resource shortage, the enterprise’s access to external resources is often limited. And some of the scattered and neglected resources within the enterprise are mistakenly seen as worthless. Therefore, effective use of the resources at hand is a challenge for enterprises, but also an opportunity. Entrepreneurial patchwork is regarded as the most effective means for new enterprises to break the constraints of resource shortage (Baker and Nelson, 2005), which can create unique values and help improve competitive advantages and performance. In addition, resource integration also plays an important role. On the one hand, resource integration can improve strategic flexibility and promote opportunity development, thereby enhancing enterprise performance (Hitt et al., 2003; Cheng and Huizingh, 2014). On the other hand, through resource integration, enterprises can achieve the consistency of resources and strategies, and also can improve the ability to cope with environmental changes and their competitive advantages (Teece, 2007; Sirmon et al., 2011). Opportunities are derived from resources, and their value also depends on their integration with the resources. Consequently, internal integration can realize opportunities through the patchwork and integration of resources with different strategic orientations in the process of starting a business, which effectively assists green entrepreneurial-oriented enterprises to obtain SCA. Based on this theory, we propose hypothesis 4.

*H4*: The positive correlation between GEO and SCA is stronger in enterprises with a higher ITI level than in enterprises with a lower ITI level.

Different from ITI, ETI includes the other two sub-dimensions. One is the integration of opportunity-discovery and resource-identification, the other is the integration of opportunity-utilization and resource-acquisition. Some scholars argued that identifying opportunities was the key to entrepreneurship. Opportunity identification helps to create competitive advantage in the early stage of development, and it is also the basis for commercializing products and services and making profits (Alvarez and Busenitz, 2001; Baron, 2006; Bakker and Shepherd, 2015). It focuses on identifying opportunities in dynamic environments, including changing market demands and industry trends, complex big data information, unique resource structures and business models, etc. However, even if the enterprise identifies more opportunities, it does not mean that it can create more value (Zahra et al., 2005; Petti and Zhang, 2011). The enterprise with a higher level of prior knowledge can identify more valuable opportunities. So the integration of opportunity identification and resource identification has a positive impact on new venture performance. In addition, effective use of opportunities and access to external resources, such as obtaining and utilizing support from government policies and external funds, building good partnerships with suppliers, and shaping a good reputation of customers. Therefore, external integration can help enterprises adopting different strategic orientations grasp business opportunities and create value in the process of entrepreneurship through effective identification of opportunities and facilitative access to resources. Besides, it assists green entrepreneur-oriented enterprises get competitive advantage efficiently. Based the theory above, we propose the hypothesis 5.

*H5*: The positive correlation between GEO and SCA is stronger in the enterprises with a higher ETI level than in enterprises with a lower ETI level.

**Figure 2:**
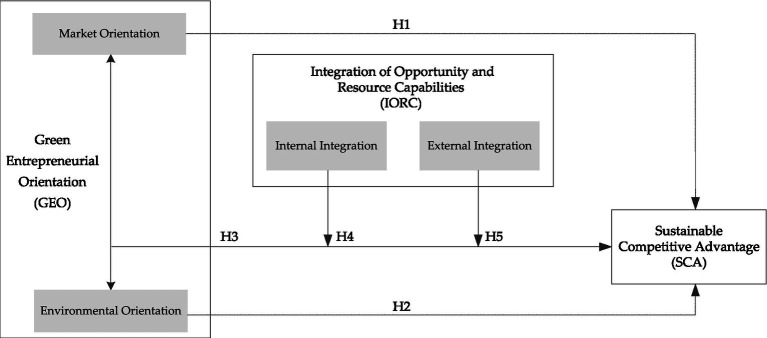
The research framework.

Figure 2 presents the overall research framework.

## Materials and methods

### Sample

To test the hypothesis proposed, this paper adopts a quantitative research method and collects data through a questionnaire survey. We developed a questionnaire based on the measurement items, which were derived from the literature, and then sent the questionnaire to the respondents. Specifically, before the formal investigation, new green enterprises (established in less than 8 years) are selected as potential samples through the two platforms of the “Statistical Yearbook of Chinese Small and Medium-sized Enterprises” and “Big Data Platform of National Small and Medium-sized Enterprises.” Through a site visit and the original questionnaire, we explored the green entrepreneurial orientation and sustainable competitive advantage of the enterprises. Then, before the formal investigation, the questionnaire design was improved and the content validity and face validity were verified by experts. Since this study revolves around the green entrepreneurship orientation and practices, we surveyed middle or senior managers of new green enterprises, they have a more comprehensive understanding in terms of their operations. And these respondents were from cities with different levels of development, including Beijing, Shanghai, Changchun, Shenyang, Hangzhou, and Shenzhen. And they were from different industries, including software and communications, manufacturing, real estate, etc. This avoids the impact of regional economic development and industry heterogeneity on the research. During the survey, we promised them the confidentiality of the information and completed the questionnaire through a face-to-face interview. The survey started from July 2017 to June 2018, and a total of 274 valid questionnaires were collected. Table 1 shows the characteristics of the collected samples.

**Table 1:** Characteristics of survey samples.

Enterprise scale		Established years		Posts of respondents	
1–10 people	16.1	1 year or less	15.7	State-owned	15.3
11–50 people	22.6	1–3 years	21.9	Foreign investment	14.2
51–100 people	21.9	3–5 years	19.3	Private	60.1
101–300 people	22.3	5–8 years	43.1	Joint venture	10.4
More than 300 people	17.1				
**Industry**			**Average turnover level in recent 3 years**		
Software and communications		10.6			
Manufacturing		23.0	Half a million or less		17.8
Real estate		10.9	Half a million to 1 million		15.7
Energy and environmental protection		10.9	1–2 million		14.6
Finance		8.0	2–3 million		15.0
Transportation, storage, and rent		10.9	3–5 million		13.9
Trade, wholesale, and retail		6.0	More than 5 million		23.0
Accommodation and catering		19.7			

The figures are percentages (%) of the sample.

### Measures

We revised the questionnaire regarding the suggestions of three experts (a scholar, an entrepreneur, and a member of a government department) to ensure that the measurement dimension of each item in the questionnaire was scientific and clear to the respondents. The questionnaire uses a five-point scale, with “1–5” indicating “respondents strongly disapprove” to “strongly approve” respectively.

GEO is an interactional composite orientation of MO and EO. MO is defined as the process by which an enterprise realizes its competitive advantage through competition and other means in the target market, reflecting information acquisition and information dissemination. According to Kumar et al. (2011), we use 6 items to measure MO, including “understanding customers’ needs (MO1),” “response speed to changes in customers’ product preferences (MO2),” “assessing the impact of business changes on customers (MO3),” “tracking market trends (MO4),” “keeping abreast of important things related to customers or markets (MO5),” and “keeping abreast of important things about competitors (MO6).” EO emphasizes the consideration of the environment in the process of strategy formulation. According to Chan et al. (2012), EO can be divided into internal and external environmental orientations. We use 4 items to measure internal environmental orientation (IEO), that is “cultivating employees’ awareness of environmental protection (IEO1),” “making clear policies (IEO2),” “employees’ emphasis on environmental protection (IEO3),” and “taking environmental protection as the core value (IEO4).” And we use 4 items to measure external environmental orientation (EEO), including “business is affected by changes in the natural environment (EEO1),” “the financial situation is affected by changes in the natural environment (EEO2),” “its survival is affected by changes in the natural environment (EEO3),” and “stakeholders put forward requirements to protect the environment (EEO4).

The IORC is reflected by the synergies between ITI and ETI. ITI can be divided into the integration of opportunity creation and resource patchwork (IOCRP) and the integration of opportunity utilization and resource-matching (IOURM). According to Baker and Nelson (2005) and Alvarez and Busenitz (2001), we use 4 items to measure IOCRP, including “leveraging limited resources and technology at hand (IOCRP1),” “completing new project development on a limited funding or channel basis” (IOCRP2), “gathering ideas from team members” (IOCRP3), and “creating a new collaboration model based on existing partnerships” (IOCRP4). And following Sarasvathy (2009), we measure IOURM with 4 items, including “exploiting new business opportunities with existing resources (IOURM1),” “exploiting business opportunities with complementary resources (IOURM2),” “optimizing the allocation of scarce talents (IOURM3)” and “making full use of scarce resources (IOURM4).” ETI can be divided into the integration of opportunity discovery and resource identification (IODRI) and the integration of opportunity utilization and resource acquisition (IOURA). Referring to Casson and Wadeson (2007) and Shepherd and DeTienne (2005), we use 3 items to measure IODRI, including “access to new industry information (IODRI1).” “identifying new technology trends (IODRI2).” “identifying potential customer needs (IODRI3).” “tracking leaders’ actions (IODRI4)” and “tracking changes in tax benefits (IODRI5).” And the 5 items of IOURA mainly refer to the study of Timmons et al. (1994), including “use of bank loans (IOURA1)” “use of equity financing instruments (IOURA2)” “introduction of new proprietary technologies and talent (IOURA3)” “use of government assistance (IOURA4),” and “development of new partnerships (IOURA5).”

The SCA means that the enterprise can always have an advantage over its competitors. Referring to Wu (2010), we use 5 items to measure it, including “searching for market information to address risks (SCA1),” “identifying potential consumer needs to develop new projects (SCA2) “, “acquiring market information in new areas (SCA3),” and “solving current problems based on useful information (SCA4).” “improving products with customer and competitor information (SCA5).” In addition, five control variables were used in the test model.

### Common method bias

Because the questions about independent and dependent variables were asked in the same questionnaire, there is a risk of common method bias (CMB) in the responses (Podsakoff et al., 2003). To exclude CMB, Harman’s single-factor test was used. The items of all variables were analyzed by principal component analysis. Results revealed that the variance explanation rate of the unrotated first principal common factor was 31.516% (less than 40%), indicating that common method variance is not a serious deficiency in this study.

### Indicators of reliability and validity

Table 2 shows the indicators of reliability and validity used in this paper.

**Table 2:** The indicators of reliability and validity.

Indicators			Standard
Reliability		Cronbach’s	>0.7
Validity	Construct validity	Factor loading coefficient (FLC)	>0.5
		Cumulative variance explained rate (CVER)	>50%
	Convergent validity	Composite reliability (CR)	>0.7
		Average variance extracted (AVE)	>0.5

### Reliability and validity test

Table 3 shows the results of the reliability and validity tests of GEO, IORC, and SCA. Cronbach’s α test is used to determine the reliability of the items. The Cronbach’s α coefficients of the variables are all greater than 0.85, so the reliability level is acceptable. The Kaiser-Meyer-Olkin (KMO) values were all above 0.8. And the factor loading coefficient is higher than 0.7, and the cumulative variance explained rate is over 68%. Therefore, construct validity conforms to the standard. In addition, all CR values are above 0.8, and the AVE values are above 0.5. These results show that the scale is reliable and valid.

**Table 3:** Results of the reliability and validity test.

Items				FLC	Cronbach’s	CVER	Convergent validity
GEO	MO		MO1	0.880	0.939	77.027%	CR = 0.953 AVE = 0.771
			MO2	0.891			
			MO3	0.897			
			MO4	0.860			
			MO5	0.865			
			MO6	0.873			
	EO	IEO	IEO1	0.813	0.880	72.846%	CR = 0.889 AVE = 0.666
			IEO2	0.851			
			IEO3	0.834			
			IEO4	0.764			
		EEO	EEO1	0.846	0.868		CR = 0.878 AVE = 0.643
			EEO2	0.827			
			EEO3	0.808			
			EEO4	0.720			
IORC	ITI	IOCRP	IOCRP1	0.809	0.893	68.927%	CR = 0.923 AVE = 0.759
			IOCRP2	0.753			
			IOCRP3	0.801			
			IOCRP4	0.825			
			IOCRP5	0.784			
		IOURM	IOURM1	0.847	0.855		CR = 0.896 AVE = 0.632
			IOURM2	0.855			
			IOURM3	0.902			
			IOURM4	0.880			
	ETI	IODRI	IODRI1	0.842	0.931	76.808%	CR = 0.876 AVE = 0.5851
			IODRI2	0.840			
			IODRI3	0.856			
			IODRI4	0.832			
			IODRI5	0.768			
		IOURA	IOURA1	0.735	0.913		CR = 0.8965 AVE = 0.635
			IOURA2	0.814			
			IOURA3	0.720			
			IOURA4	0.857			
			IOURA5	0.849			
SCA			SCA1	0.886	0.952	84.077%	CR = 0.964 AVE = 0.841
			SCA2	0.894			
			SCA3	0.918			
			SCA4	0.942			
			SCA5	0.943			

## Results

### Descriptive statistics and correlation

The descriptive statistical results of each variable and the correlation coefficient are shown in Table 4. The mean and the standard deviation of the variables are within a reasonable range. MO and EO are in low correlation and positively correlated with SCA, which supports the hypothesis. ITI and ETI are also in low correlated. Moreover, the five control variables effectively avoid interference to the dependent variable.

**Table 4:** Results of descriptive statistics and correlation.

Variate		Enterprise size	Year established	Industry	Sales performance	Posts of respondents	IORC		GEO		SCA
							ITI	ETI	MO	EO	
Enterprise scale		1									
Established years		0.107	1								
Industry		0.031	−0.021	1							
Sales performance		0.139*	0.077	−0.121*	1						
Posts of respondents		−0.059	−0.120*	0.044	−0.031	1					
IORC	ITI	−0.042	0.013	0.077	−0.032	0.002	1				
	ETI	−0.043	0.017	0.073	−0.125*	−0.004	0.357**	1			
GEO	MO	−0.088	0.020	0.028	−0.037	−0.097	0.105	0.129*	1		
	EO	0.009	0.003	−0.079	0.070	−0.095	0.093	0.176**	0.162**	1	
SCA		−0.067	−0.011	0.058	0.032	−0.005	−0.021	0.034	0.143*	0.143*	1
Mean value		3.020	3.150	4.380	3.600	2.580	3.839	3.709	3.870	2.635	3.345
Standard deviation		1.335	1.421	2.462	1.815	0.882	0.571	0.781	0.857	0.855	0.899

*N =* 274, ^*^p < 0.05, ^**^p < 0.01.

### Multivariate linear regression models and results

Table 5 shows the results of the multivariate linear regression of GEO, IORC, and SCA. The variance inflation factor values of the four regression models are all less than 10, so there is no multi-collinearity. The benchmark model (Model 1) is used to test the relationship between the control variables and SCA. It shows that large-scale and long-established enterprises result in lower SCA, and SCA acquisition can be increasingly difficult. In Model 2, the influence path of GEO and SCA is examined. The results indicate that MO, EO, and GEO are significantly correlated with SCA. It means that enterprises implementing the GEO strategy can achieve greater SCA. We test the interaction between the variables in Model 3, and the positive interaction between MO, EO, and SCA in Model 4, H1, and H2 are supported. Compared to the results of Model 2 and Model 3, the integration of MO and EO leads to higher SCA, and H3 is supported. And Model 4 shows the synergies of the integration of MO, EO, and ITI. Therefore, GEO and ITI are significantly positively correlated with SCA, demonstrating that greater ITI further promotes the positive effect of GEO on SCA. In addition, GEO, ETI, and SCA are positively correlated, illustrating that excellent ETI further improves the significant effect of GEO on SCA. Therefore, H4 and H5 are supported. Based on the preceding analysis, the hypotheses are all supported. GEO promotes SCA, and IORC acts in a positive role.

**Table 5:** Logistics regression analysis summary.

		Dependent variable: SCA				Result
		Model 1	Model 2	Model 3	Model 4	
**Control paths**						
Enterprise size		−0.077	−0.060	−0.063	−0.033	
Year established		−0.006	0.006	0.007	−0.015	
Industry		0.067	0.046	0.049	0.055	
Sales performance		0.051	0.050	0.051	0.065	
Respondents’ posts		−0.012	0.028	0.029	−0.027	
**Main effect paths**						
GEO	MO		0.153*	0.157*	0.088	
	EO		0.133*	0.134*	0.066	
IORC	ITI			−0.064	−0.104	
	ETI			0.023	−0.028	
**Hypothesized paths**						
H1: MO			0.153*	0.157*	0.088	Support
H2: EO			0.133*	0.134*	0.066	Support
H3: MO × EO			0.239***	0.241***	0.230***	Support
H4: MO × EO × ITI					0.187**	Support
H5: MO × EO × ETI					0.259***	Support
N		274	274	274	274	
Adjusted *R*^2^		−0.008	0.073	0.070	0.189	
△*R*^2^		–	0.089	0.004	0.121	
*F* value		0.586	8.777***	0.519	20.359***	

^*^p < 0.10, ^**^p < 0.05, ^***^p < 0.01.

## Implications

### Theoretical implications

First, in the study of the relationship between strategic orientation and competitive advantage, scholars have primarily focused on a single strategic orientation and its relationship with competitive advantage. However, few researchers have investigated the relationship between composite orientation and competitive advantage (Makhloufi et al., 2021; Zhou et al., 2021). Particularly in the field of entrepreneurship and sustainable theory, how green entrepreneurs obtain SCA is little understood. In addition, GEO increases the primeval cost of enterprises. However, whether GEO will hinder an enterprise’s SCA in the long run is worthy of further study. Therefore, investigating the interaction between GEO and market orientation implies that this interaction may have unparalleled value for assessing the impact of SCA. Our research defines and measures GEO and proposes that GEO reflects the interaction between market orientation from the perspective of entrepreneurship and environment orientation from the view of sustainability. In addition, we empirically test GEO’s relationship with SCA. The results reveal that GEO is a compound orientation, and positively promotes SCA, which supports the previous work of Jiang et al. (2018). This conclusion not only compensates for the deficiency of overemphasizing economic interest in existing entrepreneurial theory but also promotes the development of green sustainable theory.

Second, we propose and construct a core measurement system for IORC in the field of entrepreneurship, which is an absolutely novel research contribution. In the study of entrepreneurship, numerous scholars base their research on the duality theory, focusing on opportunity orientation or resource orientation (Ge et al., 2016; Suki et al., 2022). However, few scholars integrate the two core elements from a systemic perspective and investigate the core context of entrepreneurship in an integrated manner (Li et al., 2021). The reason for the dearth of systematic approaches is that the complicated relationship between opportunities and resources makes it difficult for scholars to investigate a subjective context. To reflect the essence of entrepreneurship, we draw on the ideas of *“Yin-Yang Balance*” and “cultivation between internal and external” elements, systematically combining these ideas in the theory of entrepreneurship. Based on this approach, we constructed a model of IORC, which proposes that the two core elements of opportunity and resources are integrated and connected. IORC, which represents the essence of entrepreneurship, is a core competence that entrepreneurial enterprises must possess and that has innovation and research value in the field of entrepreneurship.

Third, the paper examines the role of IORC in the adjustment between GEO and SCA, which is not only systematic but also innovative research. In the literature, the core theory of entrepreneurship remains to be applied in green entrepreneurship. Green entrepreneurship possesses the essential characteristics of entrepreneurship, while continuing to belong to the category of entrepreneurship. In any case, a green enterprise must break through the constraints of limited resources to implement green entrepreneurship activities and obtain SCA. Relevant to our results, Li et al. (2021) argued that when faced with resource constraints, internal and external integration of resources and opportunities is crucial to entrepreneurial performance. In this process, enterprises must adopt a green entrepreneurial orientation and do business based on this strategic orientation. The final hypothesis tested in this paper proposed that IORC which includes ITI and ETI can effectively promote a positive relationship between GEO and SCA. As the core competence of green and sustainable entrepreneurship, IORC plays a significant part.

### Managerial implications

First, the paper proposes that GEO helps enterprises obtain SCA. Since GEO is a composite orientation, it represents the interaction between MO and EO. Therefore, the two orientations are mutually reinforcing but not exclusive, which should be noted by start-ups seeking to engage in green entrepreneurship. In the past, many enterprises have rejected green entrepreneurship in their entrepreneurial activities. Entrepreneurs have worried that activities such as green technology cultivation or the introduction and transformation of green production systems will generate more costs for their enterprises. Research has shown this concern to be unwarranted. Conversely, enterprises that have the confidence to expand their markets based on an orientation toward the natural environment and the market are more likely to gain SCA rather than enterprises with short-term strategies. Particularly, with the improvement of the market structure, the diversification of customer needs and corporate information has been continuous, and past advantages of relying on information asymmetry have proven transient. Compared with competitors who adopt composite strategies, entrepreneurship with a single orientation will still gain a competitive advantage but will inevitably fall behind or be surpassed. In addition, today, EO will increase the credibility and ethical level of the enterprise. In addition, the brand value of the enterprise will eventually increase SCA. In short, new enterprises must pay attention to the entrepreneurial process of the combination of MO and EO, which can enable startups to obtain greater SCA.

Second, we propose and construct an IORC measurement system and investigate its role in green entrepreneurship and SCA. The research results have important practical value for entrepreneurial enterprises. Since such enterprises are inherently constrained by limited resources, how to break through this constraint and maximize the value of opportunities represents the core of entrepreneurship. The opportunity and resource orientations cannot be separated or opposed in this process. The two must be matched and integrated into one. The integration of the opportunity orientation and the resource orientation makes the core of entrepreneurship clear to startup ventures, that is, they must first ensure the integration of opportunities and resources. In addition, it offers enterprises a way to effectively cultivate this key capability. Moreover, empirical research shows that in the process of cultivating IORC two important dimensions of competence, ITI, and ETI, help GEO enterprises obtain SCA.

Third, it necessary for the government to encourage new ventures to undertake green entrepreneurial activities. The government must support and supervise firms that engage in green entrepreneurial behavior in two ways. It should create preferential policies to favor enterprises with environmentally friendly new products, new technologies or green management models. In addition, government supervision should be implemented. Emphasis should be placed on enterprises that steal, exceed greenhouse gas emissions and produce substandard products. Particularly the governments of developing countries must improve green system standards, accelerate the speed with which green standards are adopted by society through extensive publicity efforts, or establish social norms in an informal system to promote green enterprise management. Finally, the government and relevant institutions should improve training and education for new ventures and establish training institutions to help enterprises cultivate effective IORC so that new ventures can effectively obtain SCA.

## Conclusion

Since green entrepreneurship must take both economic interest and the ecological environment into account, it is undoubtedly valuable for researchers to investigate the relationship between GEO and SCA under the dual aspects of MO and EO. Since GEO is a composite orientation and the literature does not pay much attention to it, revealing its essence and measuring it represents the most important research contribution of this paper. In addition, green entrepreneurial activities must follow the core of entrepreneurship since their essence is entrepreneurship. That is, attention must be paid to the matching and integration of opportunities and resources. Therefore, the concept of IORC proposed by this paper not only integrates the two core elements of opportunity and resource from a systemic perspective but also constructs a measurement system that includes ITI and EIT. This effort also represents a valuable contribution to the literature. Based on this approach, the project further investigates the regulatory effect of IORC on GEO and SCA. Based on our research results, we offer the following conclusions.

First, both MO and EO positively promote SCA, which suggests that even a firm’s single-orientation entrepreneurial activity contributes to achieving SCA. Additionally, GEO has a positive effect on SCA, which indicates that taking both orientations into account can further increase SCA. Therefore, enterprises must pay attention to capitalizing on opportunities and market trends to continuously innovate green products, green technology, and green management mode. This composite strategy is a more valuable approach in the new economic circumstances. Next, based on entrepreneurial theory, the study breaks through the limitations of the entrepreneurial duality theory by creatively drawing on the ideas of “*Yin-Yang balance*” and the “consideration of both internal and external” elements. This approach is used to establish a systemic perspective from which to propose the concept of IORC, which includes two sub-dimensions: ITI and EIT. This study not only compensates for a deficiency in general entrepreneurial theory but also provides a new research perspective for future research. Finally, we examine the effect of IORC on green entrepreneurship and SCA and find that both ITI and EIT increase the role of green entrepreneurship in promoting SCA. That is, enterprise IORC is of substantial significance, particularly for green startups, for which it is advisable to focus on the patching together and integration of limited resources in ITI while seeking to identify and develop green opportunities in ETI.

Although our study integrates the theories of sustainable development and entrepreneurship and reveals the importance of green entrepreneurship in today’s economy, several limitations remain. First, the sample studied in the paper consists of Chinese green start-ups. Using data from a single country makes it difficult to generalize our conclusions. Future research should expand the scope of the sample. Secondly, the IORC measurement system represents promising but immature research in the field of entrepreneurship. It is necessary to revise and perfect this theory in the future. Finally, to measure the non-financial performance indicators of SCA, we focus on the evaluation and response of customers to enterprise products. Actually, the evaluation of enterprise’s products and strategies by the other stakeholders is also very influential. In follow-up research, the validity of the theoretical model proposed in this paper must be tested for other SCA indicators.

## Data availability statement

The raw data supporting the conclusions of this article will be made available by the authors, without undue reservation.

## Ethics statement

Ethical review and approval was not required for the study on human participants in accordance with the local legislation and institutional requirements. The patients/participants provided their written informed consent to participate in this study. Written informed consent was obtained from the individual(s) for the publication of any potentially identifiable images or data included in this article.

## Author contributions

WL: designing. YS: writing. YG: method and revising. All authors contributed to the article and approved the submitted version.

## Funding

This work was supported by “The 13th Five-Year” Science and Technology Project of Jilin Provincial Department of Education (Grant No. JJKH20200136KJ).

## Conflict of interest

The authors declare that the research was conducted in the absence of any commercial or financial relationships that could be construed as a potential conflict of interest.

## Publisher’s note

All claims expressed in this article are solely those of the authors and do not necessarily represent those of their affiliated organizations, or those of the publisher, the editors and the reviewers. Any product that may be evaluated in this article, or claim that may be made by its manufacturer, is not guaranteed or endorsed by the publisher.

## Appendix: Interview questions

The questionnaire uses a five-point scale, with “1–5” indicating “respondents strongly disapprove” to “strongly approve” respectively.

1. Green entrepreneurship orientation.

We are very active in identifying customer needs.

We can respond quickly to changes in customer product preferences.

We evaluate the impact of business changes on customers actively.

We regularly track market trends.

We keep abreast of important issues related to customers or the market.

We keep abreast of important things about its competitors.

We focus on cultivating employees’ environmental awareness.

We have a clear environmental policy.

Every employee attaches great importance to environmental protection.

The company regards environmental protection as its core value.

The business is affected by changes in the natural environment.

The financial position is affected by changes in the natural environment.

The company’s survival is affected by changes in the natural environment.

Stakeholders put forward requirements to protect the environment.

2. Integration of opportunity and resources capabilities.

The company is good at making use of the limited resources and technology at hand.

We can develop new projects with limited capital or channels.

We often listen to team members and collect their opinions.

New models of collaboration were created based on existing partnerships.

Existing resources can be exploited to develop new business opportunities.

Complementary resources can be exploited to develop new business opportunities.

We optimize the allocation of scarce talent.

Scarce resources can be fully utilized.

New industry information can be acquired.

New technological trends can be accurately identified.

Potential customer needs can be accurately identified.

We always track the actions of industry leaders.

We always track changes in tax benefits.

We can use bank loans to realize opportunities.

We are good at using equity financing instruments.

We introduce new know-how and talents according to strategic arrangement.

We make good use of government support.

We are good at developing new partnerships.

3. Sustainable competitive advantage.

We can effectively search and utilize market information when faced with risks.

We are able to develop new projects to meet the potential consumer demand.

We have access to market information in new areas.

We can solve current problems based on useful information.

We can improve products based on customer and competitor information.

*Rest of questions depend on the interview between researchers and respondents.
